# A case of ascending colon cancer accompanied with tumor thrombosis in the superior mesenteric vein treated with right hemicolectomy and greater saphenous vein grafting

**DOI:** 10.1016/j.ijscr.2018.09.007

**Published:** 2018-09-09

**Authors:** Shintaro Akabane, Shoichiro Mukai, Hiroyuki Egi, Tomohiro Adachi, Masatoshi Kochi, Koki Sato, Yusuke Sumi, Ikki Nakashima, Kazuhiro Taguchi, Haruki Sada, Akira Ishikawa, Wataru Yasui, Hideki Ohdan

**Affiliations:** a Department of Gastroenterological and Transplant Surgery, Applied Life Sciences, Institute of Biomedical & Health Sciences, Hiroshima University, Japan; b Department of Molecular Pathology, Institute of Biomedical and Health Sciences, Hiroshima University, Japan

**Keywords:** SMV, superior mesenteric vein, IMV, inferior mesenteric vein, CT, computed tomography, CEA, carcinoembryonic antigen, CA 19-9, cancer antigen 19-9, PET, positron emission tomography, FDG, fluorodeoxyglucose, SUV, standardized uptake value, ICV, ileocolic vein, Tumor thrombosis, Colorectal cancer, Greater saphenous vein grafting, Case report

## Abstract

**Introduction:**

The occurrence of colorectal cancer with tumor thrombosis in the mesenteric vein is very rare. Here, we report a case of ascending colon cancer with tumor thrombosis in the superior mesenteric vein (SMV) that was treated by complete resection.

**Presentation of case:**

A 48-year-old woman was initially admitted due to anemia. Ascending colon cancer coinciding with tumor thrombosis in the SMV was detected. Right hemicolectomy, tumor thrombectomy, and greater saphenous vein grafting of the SMV were performed. She underwent neoadjuvant chemotherapy with capecitabine plus oxaliplatin and did not have any recurrence.

**Discussion:**

Due to the high incidence of liver metastasis, the presence of venous tumor thrombosis may influence the patient’s length of survival.

**Conclusion:**

Complete resection of the primary cancer with tumor thrombosis and systemic chemotherapy should be considered for better prognosis.

## Highlights

• The occurrence of colorectal cancer with tumor thrombosis in the mesenteric vein is very rare.
• Complete resection of the primary cancer with tumor thrombosis is essential.
• Combined surgery and chemotherapy should be performed to prevent recurrence.

## 1 Introduction

Colorectal cancer accompanied with tumor thrombosis in the superior/inferior mesenteric vein (SMV/IMV) is quite rare [1]. Otani et al. in their review article showed that venous tumor thrombosis may be a strong risk factor for the development of liver metastasis [2]; hence, the presence of venous tumor thrombosis may influence the patient’s survival. Surgical thrombectomy and complete resection of the primary cancer should be considered for a better prognosis [1,3]. We present a case of tumor thrombosis in the SMV that was treated with right hemicolectomy and greater saphenous vein grafting. To the best of our knowledge, this is the first case report of venous tumor thrombosis due to colorectal cancer that was treated by vein grafting. This work has been reported in line with the SCARE criteria [4].

## 2 Presentation of case

A 48-year-old woman was initially admitted to a nearby hospital due to anemia. Total colonoscopy and computed tomography (CT) revealed ascending colon cancer accompanied with tumor thrombosis in the SMV. Subsequently, the patient was referred to our department for surgical treatment.

Her medical history was unremarkable. Complete blood count showed white blood cell count and hemoglobin level of 7850/μl and 12.1 g/dL, respectively. Serum biochemistry test results were as follows: aspartate aminotransferase, 17 U/L; alanine aminotransferase, 19 U/L; total bilirubin, 0.1 mg/dL; carcinoembryonic antigen, 341.9 ng/mL; cancer antigen 19-9, 3 U/mL; and C-reactive protein, 0.18 mg/dL. The coagulation marker D-dimer level was 0.7 μg/mL.

**Fig. 1:**
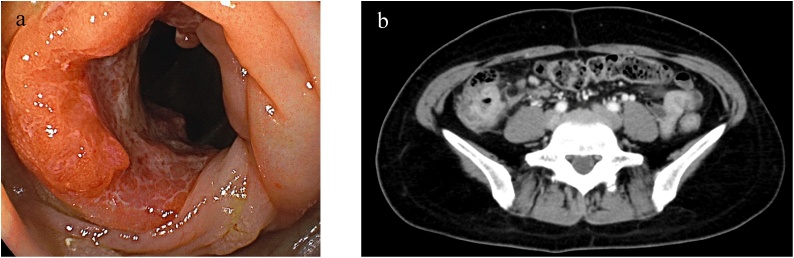
Images of the colon tumor. (a) Total colonoscopy reveals a type 2 tumor in the ascending colon. (b) Computed tomography (CT) shows an enhanced mass in the ascending colon without distant metastasis.

**Fig. 2:**
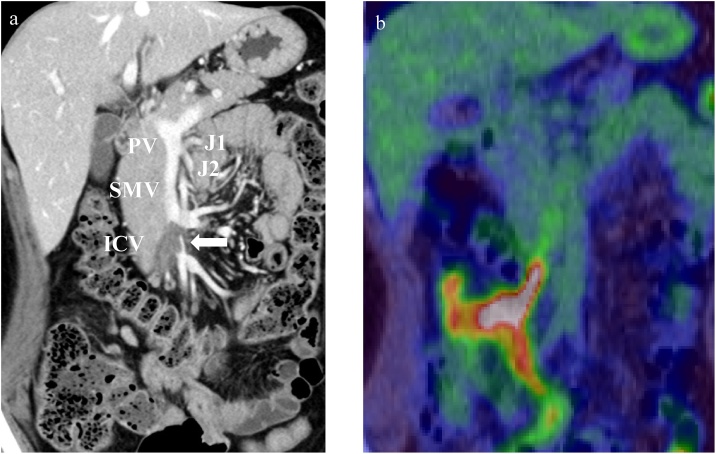
Images of the tumor thrombosis. (a) Contrast-enhanced CT shows an intraluminal filling defect from the ileocolic vein to the superior mesenteric vein (SMV; white arrow). (b) On PET-CT, abnormal FDG uptake extended from the ileocolic vein (ICV) to the SMV (SUV-max: 9.4).

Total colonoscopy revealed a type 2 tumor in the ascending colon, and moderately differentiated adenocarcinoma was diagnosed by examining the biopsy specimen (Fig. 1a). Computed tomography (CT) showed an enhanced mass in the ascending colon and an intraluminal filling defect from the ileocolic vein to the SMV (Figs. 1b and 2a). On positron emission tomography-computed tomography (PET-CT), abnormal fluorodeoxyglucose (FDG) uptake was observed in the ascending colon (SUV-max: 23.1). Abnormal FDG uptake extended from the ileocolic vein to the SMV (SUV-max: 9.4) (Fig. 2b).

**Fig. 3:**
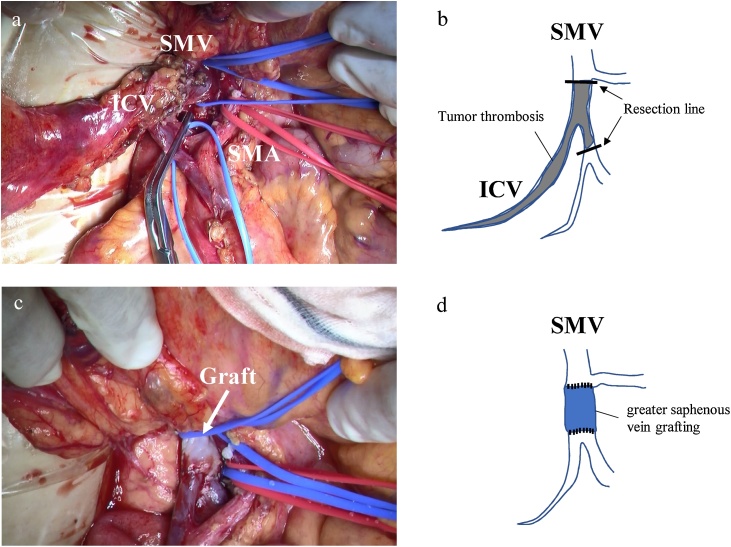
Intraoperative findings. (a) Tumor thrombosis extended from the ileocolic vein (ICV) to the root of the SMV. (b) Scheme of the tumor thrombosis. (c) After ligation of ICV and thrombectomy, greater saphenous vein grafting was performed. (d) Scheme of the vein grafting.

The patient was diagnosed with ascending colon cancer with tumor thrombosis in the SMV. The preoperative staging was T4aN1M0, stage IIIB (TNM classification). After admission, preoperative pharmacological prophylaxis was initiated with unfractionated heparin (15,000 unit/day) to prevent extension of thrombosis. Surgical resection was performed on the sixth inpatient day. Tumor thrombosis extended from the ileocolic vein to the root of the SMV. Right hemicolectomy with D3 lymphadenectomy, tumor thrombectomy of the SMV, and greater saphenous vein grafting were performed (Fig. 3). The duration of surgery was 334 min, and the blood loss was 210 mL.

**Fig. 4:**
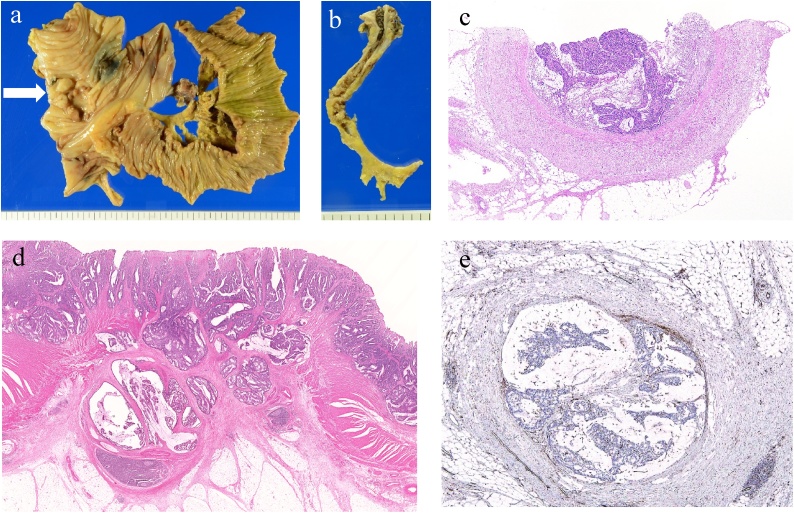
Pathological findings of the resected specimen. (a) Macroscopic findings of the ileum and ascending colon. Tumor is indicated by a white arrow. (b) Resected specimen of the ICV. (c) Hematoxylin and eosin (HE) staining of the ICV shows intraluminal invasion of moderately differentiated adenocarcinoma. (d) HE staining of a colon tumor shows a moderately differentiated adenocarcinoma reaching the subserosal layer. (e) Venous involvement (v2) is confirmed (CD 31) by immunohistochemistry.

**Fig. 5:**
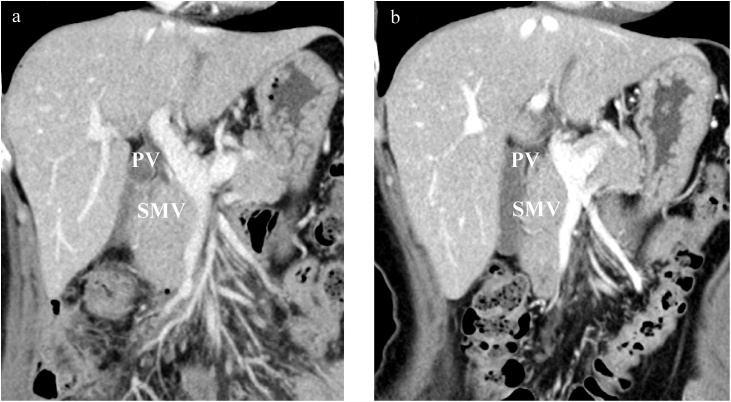
Postoperative findings. (a) The patency of the reconstructed vein was sustained and remnant tumor thrombosis was not detected on the seventh postoperative day. (b) No recurrence has been confirmed at 17 months follow-up CT imaging.

Pathological examination showed that the tumor was a moderately differentiated adenocarcinoma of the ascending colon, which reached the subserosal layer, with no lymph node metastasis (0/35) or lymphatic duct involvement (ly0) but with venous involvement (v2) (Fig. 4). The pathological staging was T3N0M0, stage IIA (TNM classification). Contrast-enhanced CT was performed to confirm patency of the reconstructed vein on the seventh postoperative day. Remnant tumor thrombosis or vein stricture was not detected in the CT image (Fig. 5a). The postoperative course was uneventful, and the patient was discharged on the eighth postoperative day. Eight courses of chemotherapy with capecitabine plus oxaliplatin were initiated 6 weeks postoperatively and continued for 6 months. Currently, 17 months have passed after the surgery, and no recurrence has been detected to date (Fig. 5b).

## 3 Discussion

Intraluminal tumor thrombus in the mesenteric vein originating from colorectal cancer is a rare condition [1]. Sato et al. reported that the incidence of advanced colorectal carcinoma with venous tumor thrombosis was 1.7% [5]. In contrast, tumor thrombosis in the portal vein system is a frequent complication of hepatocellular carcinoma because of its hypervascularization [1]. Otani et al. reported that the number of reports on venous tumor thrombosis is increasing owing to the advancement of diagnostic imaging technology [2]. 18F-FDG-PET may be useful for the differential diagnosis between tumor thrombus and venous thrombus [6].

Table 1 shows the reported cases of colon cancer accompanied with tumor thrombosis in the SMV and IMV in the Japanese population [3,[7], [8], [9], [10], [11], [12], [13], [14], [15]]. Including our case, seven and five cases in the SMV and the IMV, respectively, have been reported. Based on the reported cases, the ascending colon and the rectum were the most common tumor sites developing tumor thrombosis. With regard to SMV tumor thrombosis, the most common histological type was moderately differentiated adenocarcinoma (five of seven cases). After surgical treatment, liver metastatic recurrence occurred in four of 12 cases (25%). Given this, venous tumor thrombosis is likely to increase the risk of liver metastasis from primary colorectal cancer. Considering micrometastasis, adjuvant chemotherapy should be performed even after complete surgical tumor resection [2,13].

**Table 1:** Reported cases of colon cancer accompanied with tumor thrombosis in the mesenteric veins.

**case**	Age/Sex	location	histologic type lymphovascular invasion	T	N	M	Stage	Surgical treatment	treatment for tumor thrombosis	adjuvant chemotherapy	recurrence	prognosis	year	First Author
1	50/F	A	moderately differentiated adenocarcinoma	N.D.				right hemicolectomy	partial resection of SMV	oral 5-FU	liver, lung, pelvis	dead (5 M)	1993	Tomono
2	68/M	S, Ra	moderately differentiated adenocarcinoma ly3, v2	N.D.				low anterior resection	ligation of IMV	(–)	(–)	alive (24 M)	2000	Fujii
3	78/F	A	poorly differentiated adenocarcinoma ly3, v3	3	3	0	IIIb	right hemicolectomy, partial resection of duodenum and small bowel	removal of tumor thrombosis from SMV incision	5-FU/LV	liver metastasis (4 M)	dead (5 M)	2007	Kawashima
4	68/M	T	moderately differentiated adenocarcinoma ly2, v3	4a	1	0	IIIa	right hemicolectomy	removal of tumor thrombosis from SMV incision	FOLFOX4, UFT/LV	(–)	alive (24 M)	2009	Kanzaki
5	66/F	A	moderately differentiated adenocarcinoma ly2, v3	4b	1	0	IIIa	right hemicolectomy, partial resection of duodenum	partial resection of SMV	FOLFIRI	(–)	alive (22 M)	2009	Yamagami
6	66/M	Ra	moderately differentiated adenocarcinoma ly1, v3	3	1	0	IIIa	low anterior resection	ligation of IMV	mFOLFOX	(–)	alive (6 M)	2012	Nasu
7	54/F	Ra	well differentiated adenocarcinoma ly1, v2	3	0	0	II	low anterior resection	ligation of IMV	TS-1, FOLFOX4+Bev	liver (8 M) lung (33 M)	alive (50 M)	2012	Jimi
8	70/F	A	well differentiated adenocarcinoma ly1, v1	4b	1	0	IIIa	right hemicolectomy, partial resection of duodenum and small bowel	partial resection of SMV	FOLFOX4	(–)	alive (9 M)	2015	kamata
9	69/F	Rs	well differentiated adenocarcinoma ly1, v3	3	1	0	IIIa	low anterior resection	ligation of IMV	mFOLFOX6	lung (18 M)	alive (36 M)	2015	Matsumura
10	67/M	S	well differentiated adenocarcinoma ly1, v3	3	1	1	IIIa	sigmoidectomy, liver resection	ligation of IMV	mFOLFOX6+Bev	(–)	alive (7 M)		
11	60/F	A	moderately differentiated adenocarcinoma ly3, v3	4b	0	0	II	partial resection of transverse colon and small bowel	removal of tumor thrombosis from SMV incision	(–)	liver metastasis and dissemination	dead (21 M)	2016	Tajima
Our case	48/F	A	moderately differentiated adenocarcinoma ly0, v2	3	0	0	II	right hemicolectomy	greater saphenous vein grafting of SMV	CapeOx	(–)	alive (17 M)	2018	

M, male ; F, female ; N.D., not described A, ascending colon; T, transverse colon; S, sigmoid colon; Rs, rectosigmoid; Ra, rectum above the peritoneal reflection 5-FU, fluorouracil; LV, leucovorin; UFT, tegafur-uracil; Bev, Bevacizumab FOLFOX, oxaliplatin/5-FU/leucovorin; FOLFIRI, irinotecan/5-FU/leucovorin; CapeOx,capecitabine/oxaliplatin.

Before surgical treatment, accurate assessment of the range of tumor thrombosis and collateral circulation in contrast-enhanced CT is necessary [8]. Initial ligation of the center side vein may be essential to prevent migration of tumor thrombosis [2,7].

In the surgical treatment of tumor thrombosis in the SMV, which is a requisite drainage vein of the small bowel, we need to consider short bowel syndrome [13]. We were able to perform thrombectomy close to the root of the SMV using the greater saphenous vein graft. Massive small bowel resection was avoided by this procedure. To the best of our knowledge, this is the first case report of venous tumor thrombosis due to colorectal cancer treated by vein grafting.

## 4 Conclusion

Here, we present a case of ascending colon cancer accompanied with tumor thrombosis in the SMV that was treated with right hemicolectomy and greater saphenous vein grafting. In cases of venous tumor thrombosis, a well-planned surgical strategy and systemic chemotherapy are required for a better prognosis.

## Conflicts of interest

None of the authors has anything to disclose.

## Sources of funding

None of the authors has anything to disclose.

## Ethical approval

All procedures used in this research were approved by the Ethical Committee of Hiroshima University Hospital.

## Consent

Written informed consent was obtained from the patient for the publication of this case report and any accompanying images. A copy of the written consent form is available for review by the Editor- in-Chief of this journal.

## Author contributions

Akabane, Mukai, Egi and Ohdan were responsible for the conception and design of this study. Akabane wrote the manuscript and literature search. Adachi, Kochi, Sumi, Nakashima, Taguchi, Sada and Sato participated in the data acquisition. Akabane, Mukai, Adachi, Tamaru and Egi treated and observed the patient. Ishikawa and Yasui performed the pathological analysis. Mukai and Ohdan coordinated the study and critically revised the manuscript. All of the authors read and approved the final manuscript.

## Research registry

N/A.

## Guarantor

Shoichiro Mukai.

## Provenance and peer review

Not commissioned, externally peer-reviewed.
